# Enzyme-Triggered In Situ Assembly of Fe_3_O_4_ Nanozyme Synthesis Enables Portable Point-of-Care Detection of Acid Phosphatase

**DOI:** 10.3390/bios16060337

**Published:** 2026-06-15

**Authors:** Jianjun Kang, Yuanchun Chen, Zongcheng Shu, Cuimin Wu, Fang Ke

**Affiliations:** 1 Fujian Provincial Key Laboratory of Natural Medicine Pharmacology, Institute of Materia Medica, School of Pharmacy, Fujian Medical University, Fuzhou 350004, China; kangjianjun@fjmu.edu.cn (J.K.); shuzongcheng@fjmu.edu.cn (Z.S.); 2 Department of Gastroenterology, Fuzhou University Affiliated Provincial Hospital, Fuzhou 350001, China; syj001@fzu.edu.cn

**Keywords:** acid phosphatase, Fe_3_O_4_ nanozymes, peroxidase, colorimetric assay, smartphone detection

## Abstract

Acid phosphatase (ACP) is a clinically important enzyme whose early-stage detection is hindered by its extremely low abundance, nonspecific tissue distribution, and rapid loss of activity under conventional analytical conditions. Herein, we present a target-driven in situ nanozyme synthesis strategy that enables rapid and ultrasensitive point-of-care testing (POCT) of ACP. In this approach, ACP catalyzes the hydrolysis of L-ascorbic acid 2-phosphate sesquimagnesium (AAPS), producing ascorbic acid (AA). The generated AA partially reduces Fe^3+^ ions to Fe^2+^, thereby initiating alkaline co-precipitation and in situ formation of Fe_3_O_4_ nanoparticles. Polyvinylpyrrolidone (PVP) stabilizes the nanoparticles and preserves catalytic accessibility, while their intrinsic magnetism allows for efficient magnetic separation to eliminate matrix interference. The resulting Fe_3_O_4_@PVP nanozymes display pronounced peroxidase-like activity, catalyzing hydrogen-peroxide-mediated oxidation of 3,3′,5,5′-tetramethylbenzidine (TMB). Quantitative readout can be achieved using either spectrophotometric analysis or smartphone imaging. The sensing platform achieves a detection limit of 0.021 U/L within 40 min and demonstrates excellent sensitivity, selectivity, and operational robustness. Successful validation in human serum confirms its clinical feasibility, while smartphone-based imaging enables portable and low-cost quantification suitable for decentralized diagnostics. Collectively, this work establishes a generalizable paradigm for target-triggered nanozyme generation aimed at detecting low-abundance and labile biomarkers.

## 1. Introduction

Acid phosphatase (ACP) is a lysosomal phosphomonoesterase that hydrolyzes phosphate esters under mildly acidic conditions (pH 4–6), maintaining intracellular phosphate homeostasis and regulating metabolic pathways [1]. In mammals, ACP is mainly secreted by the prostate, bone, and liver, contributing to lysosomal turnover and extracellular phosphate metabolism [2,3,4,5]. Beyond its physiological functions, ACP has been implicated in tumorigenesis, particularly in prostate cancer, where its dysregulation promotes oncogenic signaling, proliferation, angiogenesis, and apoptosis resistance [6,7,8,9]; however, its clinical utility for early diagnosis is limited due to extremely low basal serum levels, with significant elevation typically occurring only in advanced or metastatic disease. This intrinsic scarcity, combined with inherent enzymatic instability during sample handling, poses a major analytical challenge for early detection [10,11,12,13,14]. Moreover, the lack of rapid point-of-care testing (POCT) platforms leads to prolonged turnaround times, delaying clinical decision making [15,16]. Therefore, developing ultrasensitive, rapid, and portable systems for accurate trace ACP quantification remains an urgent unmet need.

A broad range of analytical technologies has previously been explored for ACP determination, including electrochemical sensing, chromatographic analysis, and magnetic resonance imaging-based methods [17]. Although these techniques provide high analytical sensitivity, their clinical implementation is often constrained by sophisticated instrumentation, time-consuming sample preparation, and extended processing workflows [18,19]. More critically, prolonged ex vivo manipulation may partially inactivate ACP prior to measurement, increasing the likelihood of analytical bias or false diagnostic outcomes [20]. By contrast, colorimetric assays represent an attractive alternative owing to their operational simplicity, rapid response, and minimal equipment requirements [21]. Nevertheless, conventional single-signal colorimetric platforms remain susceptible to environmental fluctuation and matrix interference. These limitations are particularly problematic in early cancer diagnostics, where serum ACP elevation is subtle due to low tumor burden and altered metabolic activity [22,23,24]. Moreover, metabolic reprogramming in cancer cells frequently disrupts systemic amino-acid homeostasis and one-carbon metabolism, further complicating accurate biochemical quantification [25,26]. To overcome these challenges, enzyme-linked cascade amplification strategies have emerged as powerful sensing architectures. By integrating enzymatic recognition with signal amplification, these systems preserve the simplicity of colorimetric detection while substantially improving analytical sensitivity. Such approaches provide a promising framework for next-generation POCT platforms capable of detecting trace disease biomarkers.

Recent progress in nanozyme research has significantly expanded enzyme-mimetic sensing technologies. Compared with natural enzymes, nanozymes exhibit enhanced structural stability, tunable catalytic activity, and scalable synthesis. Metal-based nanomaterials are particularly advantageous because of their high density of catalytic sites, adjustable coordination environments, and efficient electron-transfer properties [27,28]. For example, Song reported a label-free enzyme-linked colorimetric assay for alkaline phosphatase, achieving a remarkably low detection limit of 0.037 U/L. In this system, enzymatic conversion of L-ascorbic acid 2-phosphate sesquimagnesium (AAPS) to ascorbic acid (AA) drives the reduction of metal ions to generate nanoparticles, effectively minimizing false positives commonly encountered in conventional assays [29]. However, systems relying on a single catalytic pathway often suffer from limited robustness. In contrast, iron-based nanozymes offer a distinct advantage by exploiting Fe^2+^/Fe^3+^ redox cycling to drive Fenton-like reactions, thereby generating reactive oxygen species and enabling multiple enzyme-mimetic functions simultaneously [30,31,32,33,34]. This multivalent catalytic behavior enhances both analytical reliability and signal amplification, highlighting the potential of iron-based nanozymes for accurate POCT diagnostics.

Despite these advances, metal nanoparticles frequently undergo aggregation under physiological conditions or fluctuating pH environments, which diminishes catalytic efficiency and sensing performance [35,36,37]. Polyvinylpyrrolidone (PVP) effectively mitigates this limitation through strong surface adsorption and steric stabilization, maintaining colloidal dispersion without blocking active sites. Parallel developments in smartphone-assisted analytics have further accelerated POCT innovation, enabling rapid digital image analysis using widely accessible mobile devices [38,39,40,41,42].

**Figure 1:**
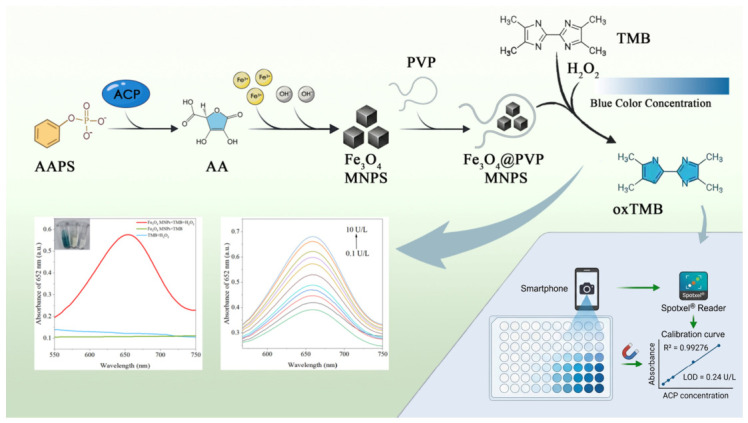
Mechanism of the ACP colorimetric assay.

Building on these concepts, we developed a target-triggered in situ nanozyme synthesis platform in which ACP functions as the specific trigger of nanozyme formation. ACP hydrolysis of AAPS generates AA, which reduces a fraction of Fe^3+^ to Fe^2+^ and drives co-precipitation of Fe_3_O_4_ nanoparticles under alkaline conditions. Importantly, the amount of AA generated is directly proportional to the ACP activity, which, in turn, determines the quantity of Fe_3_O_4_ nanozymes formed. To maintain colloidal stability and preserve catalytic performance, PVP is introduced as a stabilizing ligand to prevent nanoparticle aggregation. Furthermore, the intrinsic magnetism of Fe_3_O_4_ enables facile magnetic separation, removing untransformed substrates and potential interferents, thereby reducing background interference and further improving detection reliability. The generated Fe_3_O_4_@PVP nanozymes catalyze H_2_O_2_-mediated oxidation of TMB, producing a blue chromogenic signal quantitatively correlated with ACP concentration (Figure 1). Compared with conventional assays, this in situ strategy eliminates pre-synthesized catalysts, reduces false positives, and achieves ultrasensitive detection within 40 min. Smartphone integration further enables portable quantitative analysis, establishing a broadly applicable POCT framework.

## 2. Materials and Methods

### 2.1. Materials and Reagents

Reagents were employed directly upon receipt, with no additional purification steps performed. For all analytical experiments conducted, deionized water served as the exclusive water source. L-ascorbic acid 2-phosphate sesquimagnesium (AAPS), L-ascorbic acid (AA), polyvinylpyrrolidone (PVP), sodium hydroxide (NaOH), and ferric chloride hexahydrate (FeCl_3_·6H_2_O) were procured from Sinopharm Chemical Reagent Shanghai Co., Ltd. (Shanghai, China). 3,3′,5,5′-Tetra methylbenzidine (TMB), acid phosphatase (ACP), terephthalic acid (TPA), H_2_O_2_, L-histidine (L-His), leucine, cysteine, phenylalanine, cystine, lysine, and arginine were obtained from Aladdin Reagent Co., Ltd. (Shanghai, China). Human serums were procured from Hengyuan Biological Technology Co., Ltd. (Shanghai, China), Gemini Bioproducts, Llc. (West Sacramento, CA, USA), Thermo Fisher Scientific (Shanghai, China) Co., Ltd., and Beijing Boleide Biological Technology Co., Ltd. (Beijing, China).

### 2.2. Measurements and Characterizations

X-ray diffraction (XRD) patterns were obtained using a D8 ADVANCE X-ray diffractometer (Bruker, Karlsruhe, Germany). Scanning electron microscopy (SEM) was first used for morphological characterization, with images acquired via a Hitachi S-4800 microscope from Hitachi Ltd. (Tokyo, Japan). For optical property analysis, a UV-2600 spectrophotometer (Unico, Shanghai, China) was applied to determine the UV–visible absorption spectra. To gain deeper insights into the surface chemical composition, X-ray photoelectron spectroscopy (XPS) analysis was performed using a Thermo Scientific K-Alpha spectrometer, which was provided by Thermo Fisher Scientific Inc. (Waltham, MA, USA). Electron spin resonance (ESR) spectra were characterized by a Bruker EMXPLUS spectrometer (Bruker, Karlsruhe, Germany).

### 2.3. Synthesis of Fe_3_O_4_@PVP MNPs

To realize a target-driven in situ nanozyme synthesis strategy, a control experiment was performed using various FeCl_3_ solutions, and the resulting outcomes were examined by UV–vis absorption spectroscopy. ACP bioassays were performed in Hac-NaAc buffer (pH 4.0). AAPS solution (50 µL, 2.5 mM) was introduced into a 96-well plate, followed by the addition of ACP solutions of varying concentrations (50 µL). After incubation for 30 min to ensure complete enzymatic hydrolysis, FeCl_3_·6H_2_O solution (50 µL, 5 mM) was added, gently pipetted to mix, and incubated for one minute. Subsequently, 50 µL of 0.5 M NaOH solution was added and gently pipetted again. The enzymatically generated AA reduced a fraction of Fe^3+^ ions, initiating the formation of Fe_3_O_4_ nanoparticles under alkaline conditions. UV–vis absorption spectroscopy was used to monitor nanozyme generation.

Additionally, to exclude interference from the mixed system, Fe_3_O_4_ nanoparticles were synthesized using magnetic separation technology for subsequent experiments: Fresh stock solutions of ACP (10 U/L) and AAPS (2.5 mM) were prepared in 0.01 M HAc-NaAc (pH 4.0), and 2.5 mL of each was mixed in a 50 mL round-bottom flask, followed by incubation at room temperature with gentle stirring (300–400 rpm) for 30 min to ensure homogeneity. Under continuous stirring, 2.5 mL of 5 mM FeCl_3_·6H_2_O solution was added first, then 2.5 mL of PVP solution (0.1% *w*/*w*) dropwise, and after 2 min of stirring, 3.5 mL of 0.5 M NaOH solution was immediately added dropwise, with the reaction vigorously stirred at room temperature for 10 min. The reaction yielded 13.5 mL of a black suspension containing the synthesized Fe_3_O_4_ nanoparticles. The nanoparticles were then collected via magnetic separation, washed twice with deionized water, and redispersed in 10 mL of deionized water with uniform stirring. This step effectively removes residual free Fe^2+^/Fe^3+^ ions, unreacted AAPS, AA, and other soluble species, ensuring that subsequent catalytic measurements reflect solely the activity of the purified Fe_3_O_4_@PVP nanozyme rather than any homogeneous Fenton reactions of dissolved iron ions. The washed nanoparticle dispersion was then set aside for subsequent use.

### 2.4. Peroxidase-like Activity of Fe_3_O_4_@PVP MNPs

Colorimetric assays were employed to evaluate the peroxidase-mimetic activity of Fe_3_O_4_@PVP MNPs, with TMB used as the substrate; detailed experimental protocols for this process are provided in Table S1. Once the three mixed solutions were prepared, they were incubated at ambient temperature for a duration of 10 min. After completing the incubation step, a spectrophotometer (Section 2.2) was utilized to acquire UV–vis absorption spectra for each solution group across the wavelength range of 550–750 nm, and the corresponding absorbance values were documented simultaneously.

Additionally, ·OH production was evaluated using TPA as a fluorescent probe. TPA (2 mM) was dissolved in dimethylformamide and mixed with reaction components according to predefined compositions. Samples were incubated in the dark for 12 h before fluorescence spectra were recorded at an excitation wavelength of 315 nm.

### 2.5. Evaluation of Steady-State Kinetics of Fe_3_O_4_@PVP MNPs

To evaluate the steady-state kinetics of Fe_3_O_4_@PVP MNPs, 100 μL of Fe_3_O_4_@PVP magnetic nanoparticles (with 10 U/L of in situ-generated ACP) was mixed with 500 μL of HAc-NaAc buffer (pH 4.0), 200 μL of TMB solution at varying concentrations (0.1–2.5 mM), and 200 μL of H_2_O_2_ solution (2.5 mM). After preparing the reaction mixture, the absorbance of oxidized TMB (oxTMB) was measured at a wavelength of 652 nm using a UV–vis spectrophotometer.

Subsequently, the Lineweaver–Burk plot was employed to calculate the key Michaelis–Menten parameters, namely Km (Michaelis constant) and Vmax (maximum initial reaction rate). For clarity, the definitions of the relevant parameters are provided below: V corresponds to the initial reaction rate, Vmax represents the maximum initial rate of the reaction, [S] denotes the substrate concentration, and Km stands for the Michaelis constant.


$$\frac{1}{V}=\frac{\mathrm{Km}}{V\max}\cdot \frac{1}{\left[S\right]}+\frac{1}{V\max}$$

### 2.6. Optimization of Assay Conditions

To investigate how the microenvironment modulates nanozyme performance, we performed a comprehensive optimization of key parameters, namely pH, temperature, reaction duration, and H_2_O_2_ level. In the pH study, the Fe_3_O_4_@PVP MNP-TMB mixture was adjusted to a series of pH values spanning 2.0–7.0, held for 10 min, and then the absorbance at 652 nm was recorded with a UV–vis spectrophotometer (Section 2.2). Temperature effects were examined by holding the reaction mixtures at temperatures from 10 to 50 °C over a 10 min incubation; color development and the absorbance at 652 nm were both monitored spectrophotometrically at predetermined time points during this period. The influence of reaction time was evaluated by allowing the mixture to stand at room temperature and measuring the absorbance at 652 nm at regular intervals across a 0.5–30 min window. For H_2_O_2_ concentration, 200 μL portions of H_2_O_2_ solutions with concentrations ranging from 5 to 25 mM were introduced under ambient conditions, and the 652 nm absorbance was immediately acquired.

### 2.7. Selectivity of the Colorimetric Assay

The selectivity of the ACP-based assay was evaluated by examining its response toward a panel of amino acids, namely leucine, cysteine, phenylalanine, cystine, lysine, arginine, and histidine, along with other common interfering compounds like urea and Na^+^. Stock solutions of each potential interferent were prepared at approximately one-fold, two-fold, and five-fold of their physiological concentrations and subsequently mixed with the optimized Fe_3_O_4_@PVP MNP-TMB probe. To further probe the influence of ALP on the reaction, a 10 U/L ALP solution was first prepared. Two separate sets of experiments were then conducted following the previously described procedure: one employing the [ALP + AAPS + Fe^3+^ + H_2_O_2_ + OH^−^ + TMB] system and the other the [ACP + AAPS + Fe^3+^ + H_2_O_2_ + OH^−^ + TMB] system. The absorbance of the resulting mixtures was recorded at 652 nm. All selectivity tests were carried out in triplicate (*n* = 3) to ensure reliability.

### 2.8. Colorimetric Assay of ACP and Smartphone-Based Color Readout

For dose-response measurements, Fe_3_O_4_@PVP MNPs were generated in situ by introducing 100 μL of ACP solutions covering the concentration range 0.1–10 U/L. After magnetic separation, the reaction mixture was then supplemented sequentially with 500 μL of HAc-NaAc buffer (pH 4.0), 200 μL of H_2_O_2_ (2.5 mM), and 200 μL of TMB (0.5 mM). After incubation at 35 °C for 10 min, the absorbance spectrum from 550 to 750 nm was collected using a UV–vis spectrophotometer (Section 2.2). Each concentration was tested in triplicate (*n* = 3).

For the smartphone-assisted readout, a 200 μL aliquot of each reacted solution was transferred into the wells of a 96-well plate. Colorimetric signals were captured using a smartphone installed with the Spotxel^®^ Reader application, a freely available tool that quantifies color intensity in multi-well formats. The app was operated in blue filter mode, and the color intensity of the blue channel was extracted from the well images. By correlating this intensity with the known acid phosphatase concentrations, a quantitative calibration relationship was established.

### 2.9. Human Serum Sample Analysis

To demonstrate the practical applicability of the proposed sensing platform in ACP detection, human serum samples obtained from four independent commercial suppliers (Section 2.1) were employed as representative model matrices. Each sample was centrifuged at 10,000 rpm for 20 min, and the supernatant was diluted two-fold with ultrapure water. Subsequently, 50 μL of the diluted serum was mixed with 50 μL of AAPS solution (2.5 mM) and incubated for 30 min at room temperature. Then, 50 μL of FeCl_3_·6H_2_O (5 mM) and 50 μL of PVP solution (0.1% *w*/*w*) were added, followed by 50 μL of NaOH (0.5 M). After the in situ synthesis was complete, the generated Fe_3_O_4_@PVP nanoparticles were magnetically collected, washed twice with deionized water to remove the serum matrix, and redispersed in 500 μL of HAc-NaAc buffer (pH 4.0). The washed nanoparticle dispersion was then used in the optimized reaction system with H_2_O_2_ and TMB, and the ACP concentration was determined using the colorimetric method described above.

## 3. Results and Discussion

### 3.1. Detection of Fe_3_O_4_@PVP MNP Production

To check the experimental design, we carried out a control experiment with different FeCl_3_·6H_2_O solutions. We looked at the results with UV–vis absorption spectroscopy. Figure S1a shows that the mixture of FeCl_3_·6H_2_O, ACP, and AAPS gave a typical plasmon peak of Fe_3_O_4_ MNPs at about 309 nm after 10 min at room temperature. The Fe_3_O_4_ MNPs formed because Fe^3+^ was reduced by AA. AA was made from the enzyme reaction of ACP and AAPS. But other mixtures (Fe_3_O_4_ MNPs + ACP and Fe_3_O_4_ MNPs + AAPS) did not show the peak. These first results showed that the AA from the breakdown of AAPS could help form Fe_3_O_4_ MNPs in the reaction solution. Then we made ACP solutions with different concentrations and let them react with AAPS for 10 min. In this step, the amount of AA made depended on the ACP concentration. After we added the FeCl_3_ solution, a typical plasmonic peak appeared within 10 min. This confirmed that Fe_3_O_4_ MNPs formed. The peak positions changed with the ACP concentration (Figure S1b). When the ACP concentration was high (10 U/L), the plasmonic peak of the Fe_3_O_4_ MNPs was exactly at 309 nm [43]. When the ACP concentration was low (0.01 U/L), the peak moved to shorter wavelengths. The peak position changed because the sizes and shapes of the nanoparticles were different under different AA concentrations.

### 3.2. Characterization of Fe_3_O_4_@PVP MNPs

**Figure 2:**
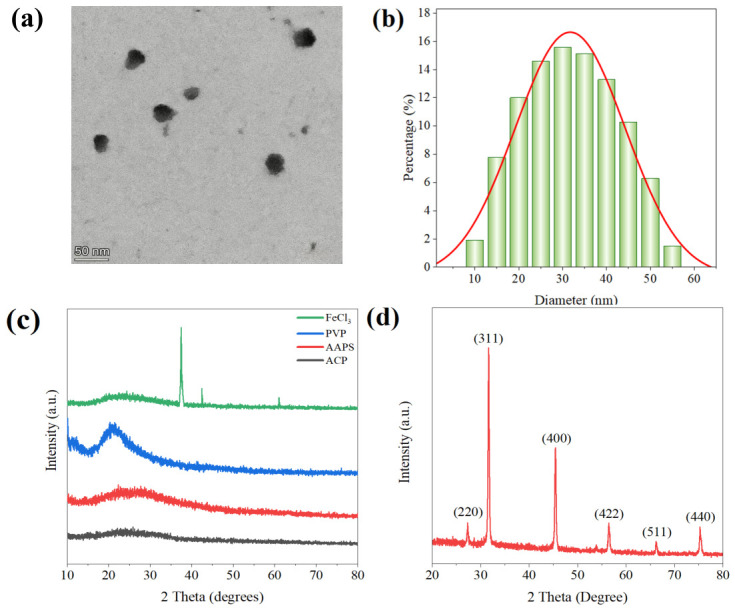
(**a**) SEM observation of the surface morphology and architecture of Fe_3_O_4_@PVP MNPs; (**b**) histogram displaying the size distribution of Fe_3_O_4_@PVP nanoparticles; (**c**) X-ray diffraction of the precursors (AAPS, PVP, FeCl_3_, and ACP); (**d**) X-ray diffractogram of the as-synthesized Fe_3_O_4_@PVP MNPs.

The morphological and structural properties of Fe_3_O_4_@PVP MNPs were characterized via SEM (Figure 2a). Correspondingly, the detailed morphological features of Fe_3_O_4_@PVP MNPs are presented in Figure 2b. It was observed that the particle size distribution of the Fe_3_O_4_@PVP MNPs ranges from 10 to 55 nm, with a higher proportion of Fe_3_O_4_@PVP MNPs falling within the 30–35 nm range.

To further confirm the in situ formation of Fe_3_O_4_@PVP nanoparticles, the reaction mixture (ACP + AAPS + FeCl_3_ + NaOH + PVP) was incubated for different time periods (2, 5, 10, 15, 20, 25, and 30 min). At each time point, the resulting nanoparticles were collected by magnetic separation, washed twice with deionized water, and immediately subjected to particle size analysis. As shown in Figure S2, the average particle diameter gradually increases from approximately 20 nm at 2 min to about 30–35 nm at 10 min, after which it remains essentially stable. This time-dependent growth directly demonstrates that nanoparticles are being formed and maturing within the reaction mixture. Furthermore, the successful magnetic collection of the nanoparticles at each time point confirms their magnetic nature, which is a characteristic property of Fe_3_O_4_. In contrast, in the control experiment without AAPS, magnetic separation yielded no detectable nanoparticulate material, and SEM imaging confirmed the absence of nanoparticle formation. These control results demonstrate that nanoparticle formation is strictly dependent on both ACP and its substrate AAPS, consistent with the proposed mechanism that ACP-catalyzed hydrolysis of AAPS generates AA, which, in turn, triggers the formation of Fe_3_O_4_@PVP nanoparticles.

The crystal structure of AAPS, PVP, FeCl_3_·6H_2_O, ACP, and Fe_3_O_4_@PVP MNPs prepared via the in situ ACP method was characterized by XRD analysis. As shown in Figure 2c, no characteristic diffraction peaks of metallic Fe_3_O_4_ are observed; only features corresponding to amorphous FeCl_3_·6H_2_O and excipients appear, confirming that FeCl_3_·6H_2_O exists as Fe^3+^ with no metallic impurities. In contrast, as shown in Figure 2d, distinct diffraction peaks at 30.1°, 35.5°, 43.1°, 53.4°, 57.0°, and 62.6° can be clearly observed, which correspond to the (220), (311), (400), (422), (511), and (440) crystal planes of Fe_3_O_4_ (JCPDS No. 19-0629), respectively. The sharp peak shapes and low baseline noise indicate good crystallinity of the prepared Fe_3_O_4_@PVP MNPs.

It is worth noting that the ACP-catalyzed hydrolysis of AAPS releases phosphate anions at mM concentration alongside AA. Theoretically, these phosphate ions could co-precipitate with Fe^2+^/Fe^3+^ to form iron (II) phosphate, iron (III) phosphate, or related hydrogen phosphate salts. However, under our strongly alkaline synthesis conditions (0.5 M NaOH), the formation of Fe(OH)_2_/Fe(OH)_3_ and their subsequent rapid co-precipitation into Fe_3_O_4_ is kinetically dominant over the nucleation and growth of iron phosphate phases. Importantly, the XRD pattern shows no characteristic diffraction peaks of crystalline FePO_4_ or Fe_3_(PO_4_)_2_, confirming that Fe_3_O_4_ is the primary crystalline product. Furthermore, even if minor amounts of amorphous iron phosphate do form, they are non-magnetic and are effectively removed during the magnetic separation step (Section 2.3) before the catalytic assay, thus posing no interference to the subsequent colorimetric detection. This is further supported by the excellent analytical performance observed in selectivity and real sample studies.

We further employed XPS to determine the valence state of the Fe_3_O_4_ species. The full survey spectrum (Figure S3) confirms the presence of Fe, C, N, and O elements. Previous reports indicate that the Fe 2p_3/2_ peak for Fe_3_O_4_ lacks a satellite peak [44]. Our results (Figure S4a) also show no such satellite peak. The binding energies of Fe 2p_3/2_ and Fe 2p_1_/_2_ are 710.6 eV and 724.1 eV, respectively. Figure S4b displays the XPS spectrum of Fe 3p for Fe_3_O_4_. The expected Fe^2+^:Fe^3+^ ratio in Fe_3_O_4_ is approximately 1:2. Using the parameters defined above, deconvolution of the peaks yields an Fe^2+^:Fe^3+^ ratio of 0.29:0.61.

To evaluate the stability of the reaction system, we compared the particle size and peroxidase activity of a 10 U/L ACP in situ-synthesized Fe_3_O_4_@PVP MNP solution before and after two months of storage at room temperature. As shown in Figure S5a,b, neither the particle size nor the peroxidase activity changed significantly after two months, indicating that the Fe_3_O_4_@PVP MNP system prepared via ACP in situ synthesis exhibits excellent physicochemical stability.

### 3.3. Peroxidase-Like Activity

Although the intrinsic peroxidase-like activity of Fe_3_O_4_ nanoparticles has been well documented since the pioneering work of Gao et al. [45] in 2007, it was necessary to functionally verify that the Fe_3_O_4_@PVP nanoparticles generated through our specific ACP-triggered in situ synthesis route retain sufficient catalytic activity under the assay conditions.

**Figure 3:**
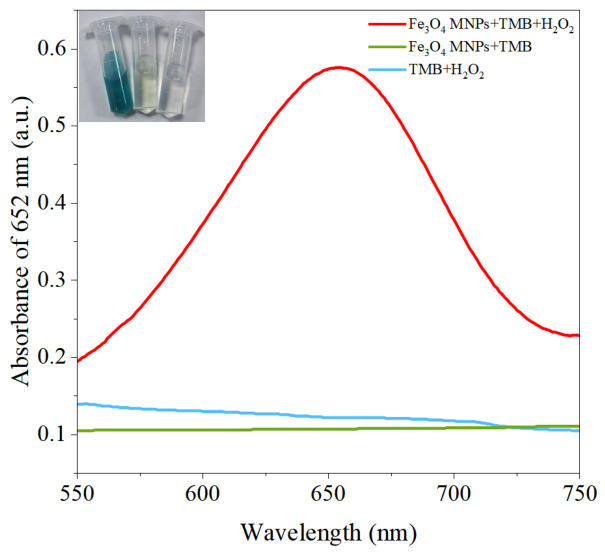
Comparison of peroxidase-mimicking activity of Fe_3_O_4_@PVP MNPs under different conditions.

All catalytic activity assays described below were performed on the Fe_3_O_4_@PVP nanoparticles after magnetic separation and washing, thus eliminating any potential interference from residual free iron ions. To characterize the peroxidase-related activity of Fe_3_O_4_@PVP MNPs, we first assessed their peroxidase-mimicking properties using the color change of TMB (common chromogenic enzyme substrate) and confirmed the results through synthesis experiments (Figure 3). The detection principle is based on the hydrolysis of AAPS catalyzed by ACP to generate AA, which subsequently reduces Fe^3+^ to form Fe_3_O_4_@PVP MNPs. To further elucidate the catalytic mechanism, a series of control experiments were performed: In the [H_2_O_2_ + TMB] system without Fe_3_O_4_@PVP MNPs, the absorption in the visible region was minimal, indicating no oxidation reaction occurred. This also confirms that no Fenton-active free iron ions were present in the system after magnetic purification, as they would otherwise have catalyzed H_2_O_2_ decomposition and TMB oxidation. Similarly, the [Fe_3_O_4_@PVP MNPs + TMB] system also showed only weak absorption, suggesting that dissolved oxygen had negligible impact and that Fe_3_O_4_@PVP MNPs themselves do not possess intrinsic peroxidase-like activity. However, when H_2_O_2_ was introduced into the [Fe_3_O_4_@PVP MNPs + TMB] system, the solution color changed markedly from colorless to deep blue, confirming that TMB oxidation took place in the [Fe_3_O_4_@PVP MNPs + H_2_O_2_ + TMB] system. These findings confirm that Fe_3_O_4_@PVP MNPs can serve as a highly sensitive colorimetric assay for the quantitative detection of ACP.

### 3.4. Steady-State Kinetics

**Figure 4:**
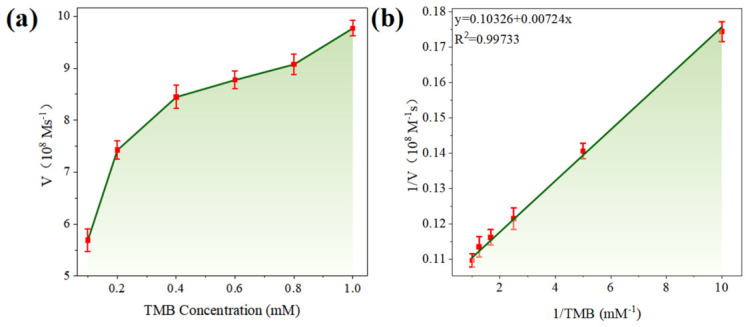
Steady-state kinetic analysis and investigation of the catalytic behavior of Fe_3_O_4_@PVP MNPs: (**a**) Michaelis–Menten saturation curve obtained with TMB as the substrate catalyzed by Fe_3_O_4_@PVP MNPs; (**b**) double-reciprocal Lineweaver–Burk plot derived for TMB oxidation mediated by the nanozymes.

To clarify the mechanism underlying Fe_3_O_4_@PVP MNPs’ peroxidase-mimetic activity, we further investigated the steady-state kinetics of TMB—its substrate—in the [TMB-Fe_3_O_4_@PVP MNPs] complex. By utilizing experimental kinetic data, we constructed a plot of initial reaction rate against substrate concentration, which yielded a typical Michaelis–Menten curve (Figure 4a). Subsequently, the Lineweaver–Burk plot (double reciprocal plot) was derived from the data presented in Figure 4b.

The calculated Vmax and Km values of Fe_3_O_4_@PVP MNPs were 9.708 × 10^−8^ M/s and 0.07 mM, respectively. Notably, its Km value was lower than those of other catalysts listed in Table S3. This finding confirms that Fe_3_O_4_@PVP MNPs exhibit strong affinity for TMB.

### 3.5. Optimization of Reaction Conditions

To detect ACP with high sensitivity and selectivity, we need to optimize key experimental conditions. These conditions include pH, incubation time, temperature, and H_2_O_2_ concentration. It should be noted that the pH-dependent oxidation of TMB is fundamentally governed by the protonation state of TMB itself, with the monoprotonated form being the reactive species susceptible to oxidation [46], as previously established. Therefore, the pH optimization in this study was performed not to reinvestigate this well-known property of TMB. Instead, we wanted to find the best pH that balances two factors. The first factor is the catalytic efficiency of the Fe_3_O_4_@PVP nanozyme, which can be influenced by its surface charge and colloidal stability at different pHs. The second factor is the chromogenic performance of TMB in our specific reaction system.

In this study, single-factor experiments and response surface methodology were further used to explore how different conditions affect the peroxidase-like activity of Fe_3_O_4_@PVP MNPs. This work had two core goals: first, to carry out enzyme kinetics research on Fe_3_O_4_@PVP MNPs, and second, to identify the optimal conditions for ACP detection. With the aim of maximizing colorimetric assay performance, each parameter was quantitatively evaluated through systematic assessment using Equation (1).

(1) $$\mathrm{Relative}\;\mathrm{Activity}=\frac{A}{A_{0}}\cdot 100\%$$

**Figure 5:**
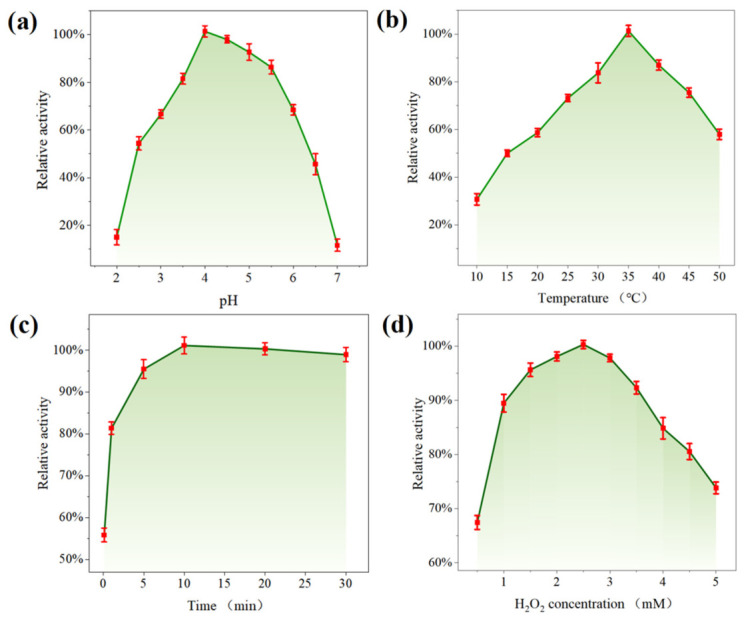
Systematic tuning of assay variables: (**a**) peroxidase-like activity profile of Fe_3_O_4_@PVP MNPs across different pH conditions; (**b**) effect of incubation temperature on the catalytic performance of the nanozymes; (**c**) time-dependent variation in the enzyme-mimetic activity; (**d**) dependence of the activity on H_2_O_2_ concentration.

Data from Figure 5a demonstrate that the relative peroxidase-like activity of the Fe_3_O_4_@PVP MNP system increased significantly as the buffer pH rose from 2.0 to 4.0, peaking at pH 4.0, and declined progressively at higher pH values. This optimal pH of 4.0 is consistent with the well-established property of TMB, whose monoprotonated form is the species prone to oxidation and predominates under mildly acidic conditions. At the same time, this pH also provides favorable conditions for the colloidal stability and surface catalytic activity of the Fe_3_O_4_@PVP nanozyme. Therefore, pH 4.0 represents the balanced optimum for the overall assay performance and was selected for all subsequent experiments.

For reaction time optimization, results from Figure 5c show that the reaction in the Fe_3_O_4_@PVP MNP-TMB system rose sharply within the first 5 min, then stabilized at the 10 min mark, and finally began to decrease slowly between 10 and 30 min. This trend suggests the reaction was nearly complete by 10 min. To further investigate the influence of H_2_O_2_ dosage on catalytic performance, we systematically examined the effects of varying concentrations of Fe_3_O_4_@PVP MNPs on the reaction system (Figure 5d).

Overall, the optimal conditions of pH 4.0, 35 °C, 10 min incubation, and 2.5 mM H_2_O_2_ were identified, with the understanding that the optimal pH primarily reflects the chromogenic properties of TMB, while the other parameters govern the catalytic performance of the Fe_3_O_4_@PVP nanozyme.

### 3.6. Mechanism Study

The catalytic pathway was further clarified by probing the formation of hydroxyl radicals (·OH) within the [Fe_3_O_4_@PVP MNPs + H_2_O_2_] system. The enzyme-mimetic activity of the Fe_3_O_4_@PVP nanozymes originates largely from a surface-driven Fenton-type reaction, mediated by the Fe^2+^/Fe^3+^ redox cycle, which accelerates the breakdown of H_2_O_2_ into highly oxidative ·OH. In this process, the continuous interconversion between Fe^2+^ and Fe^3+^ at the nanoparticle surface donates electrons to H_2_O_2_, facilitating the rupture of the O-O bond and the subsequent release of ·OH. These radicals serve as potent oxidants that convert the chromogenic substrate TMB into its blue oxTMB.

**Figure 6:**
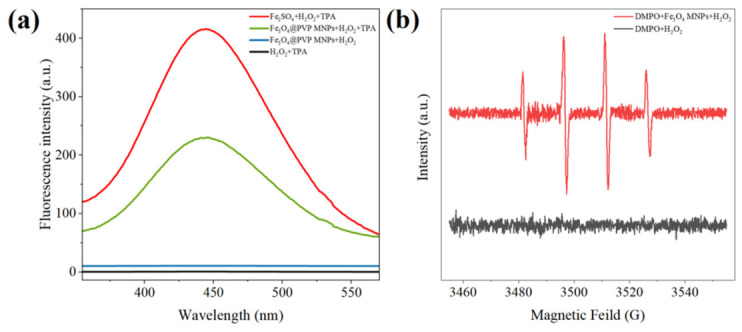
(**a**) ·OH detection via fluorescence measurement; (**b**) spin-trapping ESR analysis of ·OH.

Two complementary approaches were employed to verify ·OH production. First, fluorescence measurements revealed a distinct emission peak at 440 nm upon combining Fe_3_O_4_@PVP MNPs with H_2_O_2_ and TPA. This enhancement arises because TPA specifically captures ·OH to yield the strongly fluorescent 2-hydroxyterephthalic acid. Secondly, ESR spectroscopy with DMPO spin trapping provided direct confirmation: a characteristic 1:2:2:1 quartet pattern, typical of the DMPO-·OH adduct, was recorded in the [Fe_3_O_4_@PVP MNPs + H_2_O_2_] mixture (Figure 6b), leaving no ambiguity regarding the radical’s presence. Moreover, the fluorescence intensity scaled with the concentration of ACP used during in situ nanoparticle synthesis, with weaker signals observed at lower ACP levels. This concentration-dependent response confirms that the quantity of in situ-generated Fe_3_O_4_@PVP directly determines the yield of ·OH, thereby linking the target ACP level to the final colorimetric output. Collectively, these results demonstrate that the in situ-synthesized Fe_3_O_4_@PVP nanozymes exhibit intrinsic peroxidase-like activity by catalyzing H_2_O_2_ decomposition into ·OH, a mechanism that can be exploited for the rapid and sensitive detection of ACP.

It should be noted that because the Fe_3_O_4_@PVP nanoparticles were thoroughly washed via magnetic separation prior to these measurements, the observed ·OH generation and subsequent TMB oxidation can be exclusively attributed to the heterogeneous peroxidase-like catalysis on the nanoparticle surface, rather than to homogeneous Fenton-type reactions of any residual free iron ions.

### 3.7. Colorimetric Detection of Fe_3_O_4_@PVP MNPs

Leveraging the peroxidase-mimetic activity of Fe_3_O_4_@PVP MNP nanomaterials, an innovative detection method for ACP was developed. To achieve quantitative analysis of ACP, a detection strategy was established by monitoring the absorbance change at 652 nm (defined as ΔA = A_0_ − A, where A_0_ represents the absorbance without ACP addition and A denotes the absorbance in the presence of ACP).

**Figure 7:**
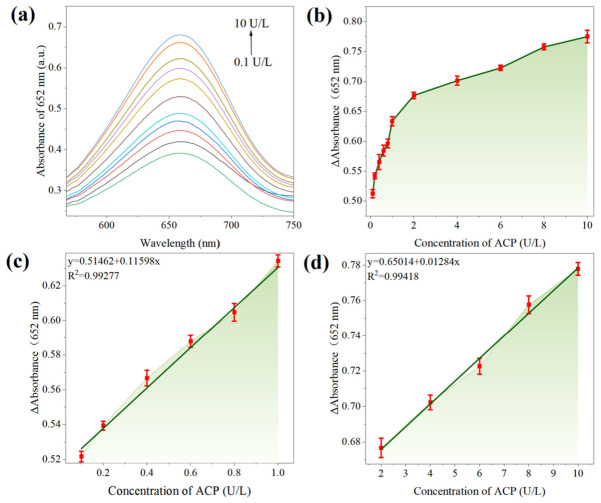
(**a**) UV–vis absorption spectra of the Fe_3_O_4_@PVP MNP-TMB system recorded at ACP concentrations of 0, 0.1, 0.2, 0.3, 0.4, 0.6, 0.8, 1, 2, 4, 6, 8, and 10 U/L; (**b**) the corresponding variation in absorbance (ΔA) plotted against ACP level across 0.1–10 U/L; (**c**,**d**) linear calibration curves established for two distinct ACP intervals, namely 0.1–1 U/L and 2–10 U/L, each demonstrating a strong linear correlation.

Under optimized experimental conditions, the introduction of ACP solutions with varying concentrations (0.1–10 U/L) into the Fe_3_O_4_@PVP MNP-TMB reaction system led to a continuous reduction in the characteristic absorption peak at 652 nm. UV–vis spectroscopic results further confirmed this phenomenon: in the absence of ACP, oxTMB exhibits a distinct absorption peak at 652 nm, while this peak gradually diminishes as ACP concentration increases (Figure 7a). This observation validates the reductive effect of ACP on oxTMB.

Regarding the linear correlation between absorbance change and ACP concentration, Figure 7b illustrates a nearly linear relationship within the 0–6 U/L range, with a more pronounced linearity in the low-concentration interval. Specifically, for ACP concentrations ranging from 0.1 to 10 U/L (Figure 7c,d), the linear fitting equation is ΔA_652_ = 0.65014 + 0.01284x (R^2^ = 0.99418). Additionally, parameters for calculating the LOD are defined as follows: LOD refers to the detection limit, Sb represents the standard error of the blank signal, and k denotes the slope of the fitted linear line.

$$\mathrm{LOD}=\frac{3\mathrm{Sb}}{k}$$

Based on the aforementioned formula, the LOD is calculated to be 0.021 U/L. As presented in Table S4, when compared with other materials used for ACP detection, Fe_3_O_4_@PVP MNPs demonstrate distinct advantages, including excellent selectivity, high sensitivity, rapid response, and simple operation procedures. These prominent merits highlight that the Fe_3_O_4_@PVP MNP-based detection method serves as a novel and efficient approach for rapid ACP detection.

### 3.8. Colorimetric Assay Selectivity

For evaluating the selectivity of ACP-induced inhibition in the Fe_3_O_4_@PVP MNP sensing system, a systematic UV–vis spectroscopic assay was performed to analyze various common amino acids and common metal ions at normal, 2×, and 5× physiological concentrations (Figure S6a,b). The results show that when interfering substances were introduced into the system, the absorbance change at 652 nm showed no significant variation compared to the blank group. This indicates that such interfering substances neither regulated the peroxidase mimicking activity of Fe_3_O_4_@PVP MNPs nor interfered with the chromogenic reaction between TMB and H_2_O_2_. In addition, we considered the selectivity of ALP for the experiment. Under the acidic conditions employed in this experiment (Figure S7), ALP activity is almost completely suppressed. Consequently, replacing ACP with ALP in the sensing platform yielded no detectable reaction, as indicated by an absorbance value close to zero. Conversely, adding ALP to the ACP-based platform did not appreciably alter the absorbance, demonstrating that ALP does not interfere with our detection system. In summary, the [Fe_3_O_4_@PVP MNPs + H_2_O_2_ + TMB] colorimetric system constructed in this study exhibits excellent selectivity and effectively avoids interference from the aforementioned potential interferents, providing reliable interference resistance for the accurate detection of ACP in subsequent practical samples.

### 3.9. Smartphone-Based Detection of ACP

**Figure 8:**
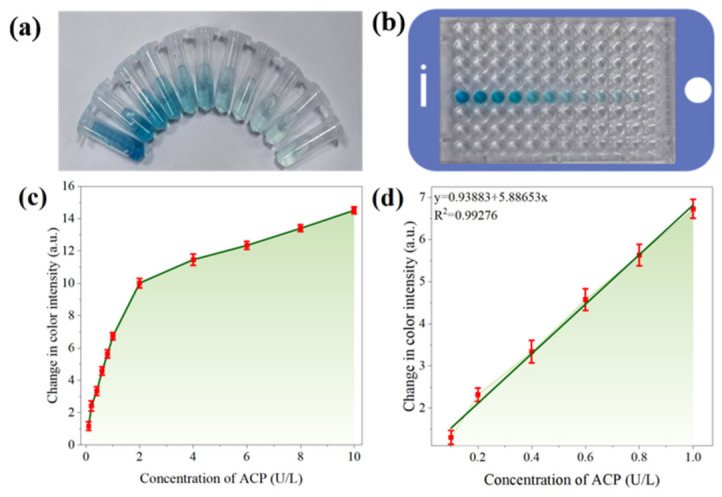
(**a**) and (**b**) Progressive color transition of the solution upon elevating the ACP concentration from 0.1 to 10 U/L; (**c**) absorbance plotted as a function of ACP level; (**d**) linear regression equation obtained over the 0.1–1 U/L concentration range.

A portable smartphone-based analytical platform was established by integrating the Fe_3_O_4_@PVP MNP-TMB colorimetric reaction for the rapid quantification of ACP. Within this system, a progressive color transition from colorless through light blue to deep blue was observed as the ACP concentration was elevated from 0.1 to 1 U/L (Figure 8a). High-resolution images of the resulting solutions at varying ACP levels were captured using a smartphone, and the corresponding color intensity at 652 nm was extracted using Spotxel^®^ software (version 2.1.6, Figure 8b). Based on these data, a calibration plot of absorbance against ACP concentration was constructed (Figure 8c), yielding a linear regression equation of y = 0.93883 + 5.88653x with an R^2^ of 0.99276 over the range 0.1–1 U/L (Figure 8d). The method exhibited a favorable linear response in this range and achieved a detection limit of 0.24 U/L. The designed smartphone-based system is operationally straightforward and cost effective, offering a dependable and practical approach for on-site ACP determination, and holds considerable promise for POCT applications.

### 3.10. Real Sample Analysis

To evaluate the applicability and precision of this method, human serum samples were analyzed, and recovery rates were determined via the standard addition approach. Table S5 presents recovery values ranging from 96.5% to 105.0%, with a relative standard deviation (RSD) below 3%. These findings suggest that the proposed approach is highly feasible for ACP quantification in diverse sample matrices. Accordingly, our results confirm that the colorimetric detection system based on the in situ Fe_3_O_4_@PVP MNP + TMB platform offers consistent and reliable performance for ACP analysis.

Furthermore, as shown in Table S6, we conducted comparative experiments between HPLC and colorimetry using human serum samples. In practical operation, HPLC involves cumbersome procedures, while colorimetry features rapid and convenient detection advantages. Additionally, there is only a minor difference in the detection results of ACP between the two methods with no statistically significant difference (*p* > 0.05). Therefore, this confirms that our detection platform can achieve rapid detection of ACP.

## 4. Conclusions

This study presents a target-driven in situ nanozyme synthesis platform for sensitive and portable detection of ACP. ACP hydrolyzes AAPS to generate ascorbic acid, which triggers the formation of Fe_3_O_4_@PVP nanozymes with intrinsic peroxidase-like activity. The system achieves a detection limit of 0.021 U/L within 40 min and integrates smartphone imaging for on-site analysis. Compared with the standard p-nitrophenyl phosphate (pNPP) colorimetric method, our approach offers three key advantages. Firstly, it provides higher sensitivity through dual signal amplification (enzymatic hydrolysis coupled with nanozyme-catalyzed TMB oxidation). Moreover, magnetic separation physically removes matrix interference, unlike pNPP where endogenous chromophores and turbidity affect readings. Lastly, the acidic working pH (4.0) suppresses alkaline phosphatase activity, ensuring enhanced selectivity. Although our multi-step design is more complex than the single-step pNPP assay, these gains in sensitivity, anti-interference capability, and selectivity justify its use for POCT in complex biological samples. This work establishes a broadly applicable framework for detecting low-abundance and labile biomarkers in decentralized diagnostics.

## Data Availability

Data are provided within the manuscript or Supplementary Information Files.

## Supplementary Materials

The following supporting information can be downloaded at: https://www.mdpi.com/article/10.3390/bios16060337/s1, Figure S1: UV-vis spectroscopic study of the formation of Fe_3_O_4_ MNPs: (a) synergistic role of ACP and AAPS and (b) effect of the concentration of ACP (0.1 U/L, 0.2 U/L, 0.4 U/L, 0.6 U/L, 0.8 U/L, 1.0 U/L); Figure S2: The particle size distribution of the [ACP + AAPS + FeCl_3_ + NaOH + PVP] system and the [ACP + FeCl_3_ + NaOH + PVP] system at different incubation times (2–30 min); Figure S3: Full XPS spectral surveys of the Fe_3_O_4_@PVP MNPs; Figure S4: (a) The XPS spectrum of Fe 2p from the fractured surface of the Fe_3_O_4_ standard sample; (b) The XPS spectrum of Fe 3p from the fractured surface of the Fe_3_O_4_ standard sample; Figure S5: (a) Comparison of particle size distribution of Fe_3_O_4_@PVP MNPs two months ago and two months of storage; (b) Comparison of peroxidase mimicking activity of Fe_3_O_4_@PVP MNPs two months ago and two months of storage; Figure S6: Comparison of inhibition of various amino acids (a) and other ion pairs (b) in the sensing system with ACP; Figure S7: The absorbance at 652 nm of [ACP + AAPS + FeCl_3_ + H_2_O_2_ + TMB] system in the presence of albumin, globulin and ALP (the four one is the [ALP + AAPS + FeCl_3_ + H_2_O_2_ + TMB]);Table S1: Solution addition method for Peroxidase-like Activity of Fe_3_O_4_@PVP MNPs; Table S2: Solution addition method for fluorescent probe TPA; Table S3: Comparison of apparent steady-state kinetic of nanophase materials; Table S4: Comparison of detection limits of ACP with those reported methods; Table S5: Detection of ACP in human serum samples (*n* = 3); Table S6: ACP analysis in real samples (*n* = 3). For references in the supplementary materials, please refer to [47,48,49,50,51,52,53,54,55,56].
