# Supplementary material for: Enzyme-Triggered In Situ Assembly of Fe3O4 Nanozyme Synthesis Enables Portable Point-of-Care Detection of Acid Phosphatase
Source: Biosensors (Basel). 2026 Jun 15;16(6):337. doi: 10.3390/bios16060337 (PMC13296935; doi:10.3390/bios16060337)
Supplement: Supplementary file 1 [file biosensors-16-00337-s001.zip › biosensors-4296250-supplementary.pdf]

Figure Information for

Enzyme-Triggered In Situ Assembly of Fe_3O_4 Nanozyme Synthesis Enables Portable Point-of-Care Detection of Acid Phosphatase

Jianjun Kang[1], Yuanchun Chen[2], Zongcheng Shu[1], Cuimin Wu[1,*], Fang Ke[1,*]

[1]*School of Pharmacy, Institute of Materia Medica, Fujian Provincial Key Laboratory of Natural Medicine Pharmacology, Fujian Medical University, Fuzhou 350004, China*

[2]*Department of Gastroenterology, Fuzhou University Affiliated Provincial Hospital, Fuzhou 350001, China*

* Corresponding author.

E-mails: kefang@mail.fjmu.edu.cn (F. Ke), wucuiming@fjmu.edu.cn (C.Wu).

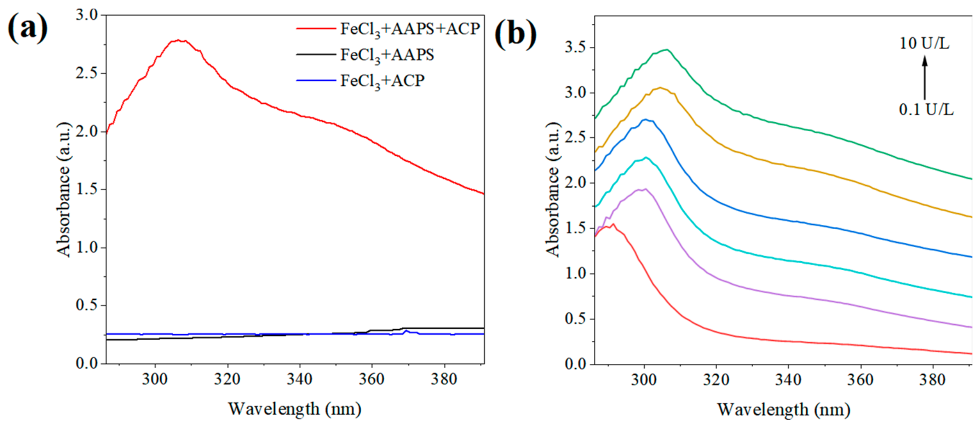

Figure S1. UV-vis spectroscopic study of the formation of Fe_3O_4 MNPs: (a) synergistic role of ACP and AAPS and (b) effect of the concentration of ACP (0.1 U/L, 0.2 U/L, 0.4 U/L, 0.6 U/L, 0.8 U/L, 1.0 U/L).

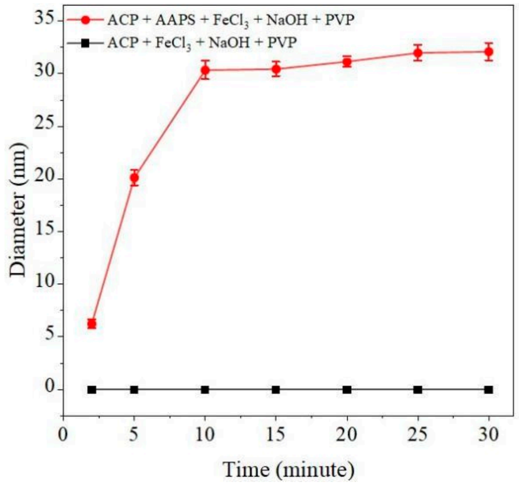

Figure S2. The particle size distribution of the [ACP + AAPS + $FeCl_3$ + NaOH + PVP] system and the [ACP + $FeCl_3$ + NaOH + PVP] system at different incubation times (2-30 min).

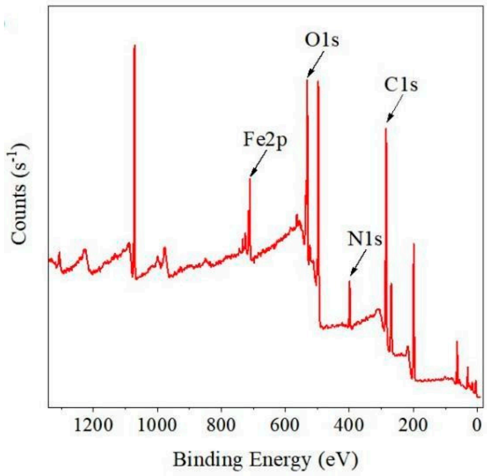

Figure S3. Full XPS spectral surveys of the Fe_3O_4@PVP MNPs.

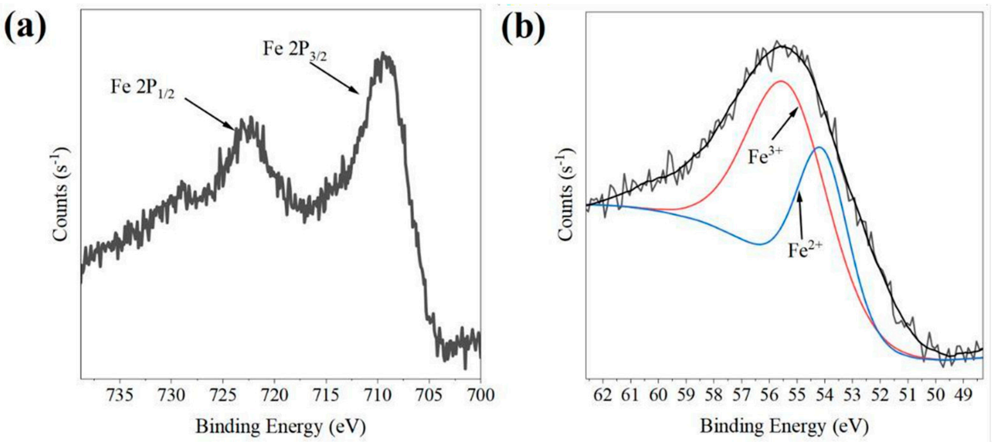

Figure S4. (a) The XPS spectrum of Fe 2p from the fractured surface of the Fe_3O_4 standard sample; **(b)** The XPS spectrum of Fe 3p from the fractured surface of the Fe_3O_4 standard sample.

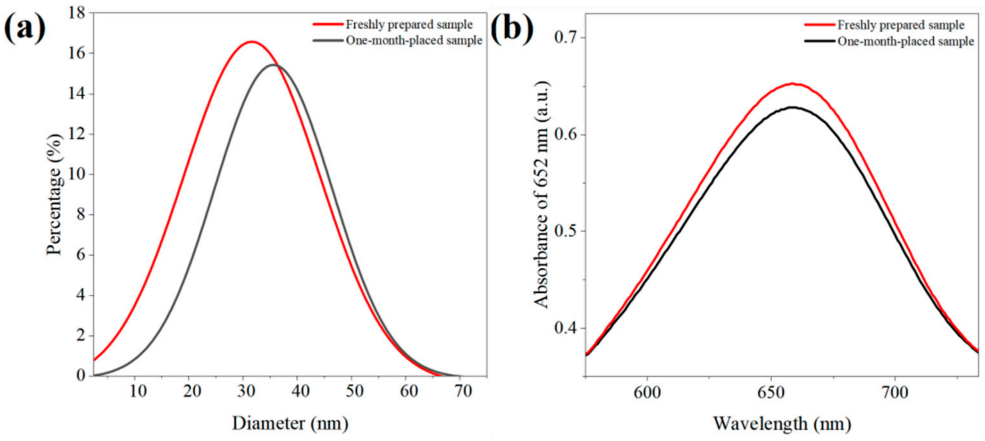

Figure S5. **(a)** Comparison of particle size distribution of Fe_3O_4@PVP MNPs two months ago and two months of storage; **(b)** Comparison of peroxidase mimicking activity of Fe_3O_4@PVP MNPs two months ago and two months of storage.

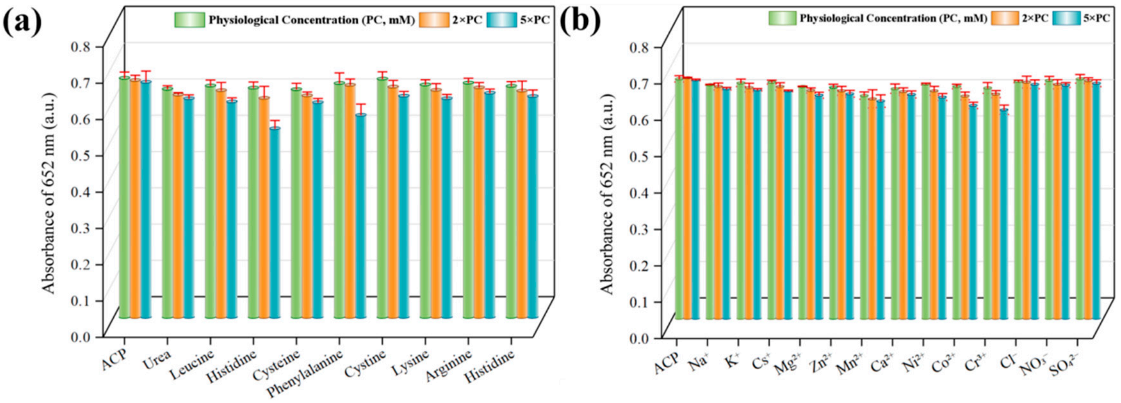

Figure S6. Comparison of inhibition of various amino acids **(a)** and other ion pairs **(b)** in the sensing system with ACP.

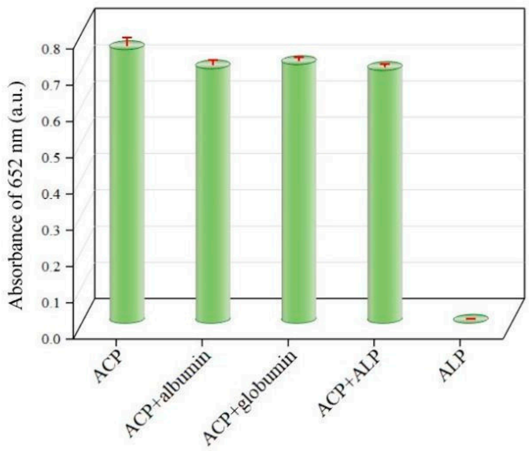

Figure S7. The absorbance at 652 nm of [ACP + AAPS + $FeCl_3$ + H_2O_2 + TMB] system in the presence of albumin, globulin and ALP (the four one is the [ALP + AAPS + $FeCl_3$ +H_2O_2 +TMB]).

Graphics Information for

Enzyme-Triggered In Situ Assembly of Fe_3O_4 Nanozymes Synthesis Enables Portable Point-of-Care Detection of Acid Phosphatase

Jianjun Kang[1], Yuanchun Chen[2], Zongcheng Shu[1], Cuimin Wu[1,*], Fang Ke[1,*]

[1]School of Pharmacy, Institute of Materia Medica, Fujian Provincial Key Laboratory of Natural Medicine Pharmacology, Fujian Medical University, Fuzhou 350004, China

[2]Department of Gastroenterology, Fuzhou University Affiliated Provincial Hospital, Fuzhou 350001, China

* Corresponding author.

E-mails: kefang@mail.fjmu.edu.cn (F. Ke), wucuiming@fjmu.edu.cn (C.Wu).

Table S1. Solution addition method for Peroxidase-like Activity of Fe_3O_4@PVP MNPs.

Group	HAc-NaAc (pH 4.0)	H_2O_2 (2.5 mM)	TMB (0.5 mM)	Fe_3O_4@PVP MNPs (10 U/L ACP in-situ generation)
Blank (μL)	500	200	200	0
Fe_3O_4@PVP MNPs (μL)	500	0	200	100
	500	200	200	100

Table S2. Solution addition method for fluorescent probe TPA.

Group	HAc-NaAc (pH 4.0)	H_2O_2 (2.5 mM)	TPA (2 mM)	$FeSO_4$ (1 mM)	Fe_3O_4@PVP MNPs (10 U/L ACP in-situ generation)
Blank (μL)	500	200	200	0	0
$FeSO_4$ (μL)	500	200	200	200	0
Fe_3O_4@PVP MNPs (μL)	500	0	200	0	100
	500	200	200	0	100

Table S3. Comparison of apparent steady-state kinetic of nanophase materials.

Nanophase materials	Substrate	K_m(mM)	$V_{max}(10^{-8}Ms^{-1})$	Reference
DNA/CuAl-LDH	TMB	1.775	4.09	[47]
TiO_2@MoS_2		2.11	5.09	[48]
Tα-MOF		3.18	2.228	[49]
2D Cu-TCPP(Fe)		1.2	0.98	[50]
CuO-Au nanoalloy		3.54	1.05	[51]
Fe_3O_4@PVP MNPs (μL)		0.07	9.708	This work

Table S4. Comparison of detection limits of ACP with those reported methods.

Method	Signal indicator	LOD(U/L)	Reference
Fluorometry	GSH-AuNCs	0.101	[52]
Fluorometry	SHCy-P	0.48	[53]
Colorimetry	Mn@HCNs	0.0851	[54]
Electrochemistry	Magneticbeads/GO	0.52	[55]
Colorimetry	CuAg nanoflowers	0.065	[56]
Colorimetry	Fe_3O_4@PVP MNPs	0.021	This work

Table S5. Detection of ACP in human serum samples (n=3).

Samples	Spiked (U/L)	Found (U/L)	Recovery (%)	RSD (%)
1#	5.32	5.59	105.0	1.0
2#	5.47	5.24	96.5	2.3
3#	6.69	6.57	98.2	2.6
4#	6.87	6.86	99.8	2.1

Table S6. ACP analysis in real samples (n=3).

Real samples	Experimental result (U/L ± SD)		RSD (%)	
	HPLC	Colorimetry	HPLC	Colorimetry
Sample 1#	0.74±0.09	0.71±0.12	1.97	2.33
Sample 2#	3.25±0.11	3.20±0.07	2.98	2.07
Sample 3#	5.56±0.14	5.52±0.15	2.45	1.46

References

47. Zhao, X.; Zhang, N.; Yang, T.; Liu, D.; Jing, X.; Wang, D.; Yang, Z.; Xie, Y.; Meng, L. Bimetallic metal-organic frameworks: enhanced peroxidase-lik e activities for th e self-activated cascade reaction. ACS Appl. Mater. Interfa ces 2021, 13, 36106-36 116. https://doi.org/10.1021/acsami.1c05615.

48. Han, L.; Meng, J.; Li, L.; Yin, X. B.; Zheng, J. Interface engineering of h ierarchical TiO_2@MoS_2@Ag for enhanced peroxidase-mimetic activity and v isual tannic acid sensing. Microchem. J. 2025, 216, 114604.https://doi.org/1 0.1016/j.microc.2025.1 14604.

49. Karami, Z.; Jeibar, A.; Sohrabi, N.; Badoei-dalfard, A.; Sargazi, G. A poro us tantalu m-based metal-organic framework (Ta-MOF) as a novel and highl y efficient perox idase mimic for colorimetric evaluation of the antioxidant capacity. Catal. Lett. 202 0, 150, 2167-2179. https://doi.org/10.1007/s10562-02 0-03137-8.

50. Zhang, H.; Yang, H.; Liu, P.; Qin, X.; Liu, G. Colorimetric quantification of sodium benzoate in food by using d-amino acid oxidase and 2D metal organic framework n anosheets mediated cascade enzyme reactions. Talanta 2022, 237, 122906. https:// d oi.org/10.1016/j.talanta.2021.122906.

51. Adeniyi, O.; Sicwetsha, S.; Mashazi, P. Nanomagnet-silica nanoparticles decorated with Au@Pd for enhanced peroxidase-like activity and colorimetric glucose sensin g. ACS Appl. Mater. Interfaces 2020, 12, 1973-1987. https://doi.org/10.1021/acsam i.9b15123.

52. Li, Q.; Gao, Y.; Liu, S.-H. Fluorometric and colorimetric quantitative analysis platfo rm for acid phosphatase by cerium ions-directed AIE and oxidase-like activity. Ana l. Bioanal. Chem. 2024, 416, 1179-1188. https://doi.org/10.1007/s00216-023-0510 3-w.
53. Cai, S.; Liu, C.; Jiao, X.; He, S.; Zhao, L.; Zeng, X. A lysosome-targeted near-infrar ed fluorescent probe for imaging of acid phosphatase in living cells. Org. Biomol. Chem. 2020, 18, 1148-1154. https://doi.org/10.1039/C9OB02188D.
54. Li, N.; Yang, F.; Li, L.; Zhang, R. Engineered nanozyme-cascade catalyzed reaction for rapid acid phosphatase detection. Microchem. J. 2024, 207, 111688. https://doi.org/10.1016/j.microc.2024.111688
55. Gan, X.; Qiu, F.; Jiang, B.; Yuan, R.; Xiang, Y. Convenient and highly sensitive elect rochemical biosensor for monitoring acid phosphatase activity. Sens. Actuators B Chem. 2021, 332, 129483. https://doi.org/10.1016/j.snb.2021.129483.
56. Sacks, D.; Baxter, B.; Campbell, B. C. V.; Carpenter, J. S.; Cognard, C.; Dippel, D.; Eesa, M.; Fischer, U.; Hausegger, K.; Hirsch, J. A.; Hussain, M. S.; Jansen, O.; Jay araman, M. V.; Khalessi, A. A.; Kluck, B. W.; Lavin e, S.; Meyers, P. M.; Ramee, S.; Rüfenacht, D. A.; Schirmer, C. M.; Vorw erk, D. Multisociety consensus quality improvement revised consensus state ment for endovascular therapy of acute ische mic stroke. Int. J. Stroke 2018, *13*, 612-632. https://doi.org/10.1177/174749301877 8713
